# Artificial intelligence for the prediction of postoperative complications in the critically ill

**DOI:** 10.62675/2965-2774.20250025

**Published:** 2025-05-04

**Authors:** Vanessa Moll, Ashish K Khanna, Piyush Mathur

**Affiliations:** 1 University of Minnesota Department of Anesthesiology Minneapolis MN United States Department of Anesthesiology, Division of Critical Care Medicine, University of Minnesota - Minneapolis, MN, United States.; 2 Department of Anesthesiology, Section on Critical Care Medicine, Atrium Health Wake Forest Baptist Medical Center Winston-Salem NC United States Department of Anesthesiology, Section on Critical Care Medicine, Atrium Health Wake Forest Baptist Medical Center - Winston-Salem, NC, United States.; 3 Department of Anesthesiology, Cleveland Clinic Cleveland United States Department of Anesthesiology, Cleveland Clinic - Cleveland, United States.

## INTRODUCTION

Postoperative mortality is the third leading cause of death worldwide, accounting for approximately 4.2 million deaths within 30 days of surgery each year (following ischemic heart disease and stroke).^(1)^ Many of these deaths follow complications such as myocardial injury after noncardiac surgery, sepsis, or acute kidney injury (AKI).^(2)^ Early identification of at-risk patients is vital to optimize intraoperative and postoperative management, enabling timely intervention to prevent or mitigate complications. However, real-time, accurate risk assessment remains challenging despite the wealth of data generated perioperatively. Artificial intelligence (AI), particularly through machine learning (ML) and deep learning (DL), offers an opportunity to bridge this gap, providing tools that enhance clinicians’ abilities to predict and manage perioperative complications. Artificial intelligence applications in critical care span various domains, including early warning systems, predictive tools, treatment recommendation models, disease phenotyping, and resource management. A key advantage of these emerging applications for perioperative care is their accuracy, systematic approach to risk identification, and capacity for timely detection. These capabilities could be transformative in the management of critically ill patients. However, even though using AI to predict postoperative complications holds considerable promise, the models have yet to be prospectively validated in diverse clinical settings before their integration into clinical practice. A 2021 systematic review revealed that the majority of AI studies in the intensive care unit (ICU) focus on predicting complications (22.2%), followed by mortality prediction (20.6%), enhancing prognostic models (18.4%), and classifying patient sub-populations (11.7%). Despite the breadth of research, prospective studies remain rare, with only 18 prospective studies among the 494 included in the review.^(3)^

## PREDICTIVE MODELS IN PERIOPERATIVE CARE

Artificial intelligence-based predictive models promise to improve care for critically ill patients by forecasting postoperative complications. Bihorac et al.^(4)^ developed an ML-based model that uses electronic health record (EHR) data to predict the risk of eight major postoperative complications, including ICU admission > 48 hours, mechanical ventilation > 48 hours, AKI, sepsis, cardiovascular complications, and mortality. The model demonstrated good predictive performance, with areas under the curve (AUC) ranging from 0.82 to 0.94. A mobile app provided surgeons with real-time probabilistic risk scores, enhancing their decision-making. Similarly, Mathis et al. combined EHR with processed intraoperative waveform data to develop ML models to predict postoperative deterioration in cardiac surgery, achieving superior performance (C-statistic: 0.709 - 0.803) compared to EHR-only models (0.641). The predictions of postoperative deterioration were a composite of low cardiac index, hypotension (mean arterial pressure < 55mmHg for > 120 minutes), use of vasopressors, and ICU admission. A tensor decomposition method was used to analyze waveform features, including electrocardiogram lead II, pulse plethysmography, and arterial waveform.^(5)^ Up to 80% of adverse events in general hospital wards are preventable. However, continuous monitoring alone is not enough—integrating AI-driven technology can enhance event prediction and enable timely intervention. Here, software using single lead continuous electrocardiogram waveform and extracted heart rate variability predicted a rapid response call better than raw vital signs.^(6)^ Large language models such as ChatGPT have been recently tried to predict postoperative complications and outcomes. Using text data from EHR and various prompting methodologies, researchers used GPT-4 Turbo (OpenAI) to predict hospital mortality and the need for postoperative ICU admission with accuracy similar to traditional ML and DL methods, achieving F1 scores of 0.81 and 0.86.^(7)^

## PREDICTIVE MODELS IN THE INTENSIVE CARE UNIT

The vast volume of ICU data can overwhelm clinicians, leading to delays, reduced care quality, or errors, all typical of the ‘information overload’ common in this environment. Integrating predictive analytics and clinical decision support systems offers the potential to enhance clinical management and improve patient outcomes. Various AI models have been developed to predict common ICU complications, such as AKI, sepsis, and ICU mortality. Meyer et al. utilized a recurrent neural network model to predict key complications such as renal failure, postoperative bleeding, and mortality in cardiac surgery patients. The model demonstrated a positive predictive value (PPV) ranging from 0.84 to 0.90 and sensitivities between 0.74 and 0.94.^(8)^ Alfieri et al. developed an ML model to predict AKI in ICU patients using data from 145 ICUs across three countries. The model achieved an AUC of 0.884, with predictions made within 14 hours of ICU admission.^(9)^ AKI predictor achieved an AUC of 0.75 *versus* 0.80 for clinicians. The authors concluded that physicians were overestimating AKI risk, and AKI predictor added value in systematic AKI risk stratification in the ICU.^(10)^

Sepsis remains a global challenge due to its high morbidity, mortality, and costs, with early detection hindered by nonspecific symptoms and a lack of definitive tests. Artificial intelligence algorithms integrating multimodal data, such as vital signs and lab results, show promise, achieving a sensitivity of 0.81 and specificity of 0.94 for shock prediction.^(11)^ This early work is promising as combining text data with vital signs and laboratory data can improve predictions of AI models in an approach similar to clinicians’ decision-making. In a systematic review and meta-analysis of 23 studies looking at prediction of early onset of sepsis in ICU, researchers found random forest (RF) and extreme gradient boosting (XGB) to be the most frequently used ones with a high degree of accuracy (accuracy = 0.911 for RF (n = 9), 0.957 for XGB (n = 7).^(12)^ However, while ML algorithms that can detect evolving sepsis earlier than rule-based methods have proliferated, limited algorithms have been implemented in clinical practice and shown improved outcomes.^(13)^ However, prediction is the first step to improving clinical management and patient outcomes. The use of pulmonary artery catheters (PAC) in critically ill patients illustrates this point. Initially, PACs were widely adopted with the expectation that continuous hemodynamic monitoring would enable the early identification of cardiovascular instability, allowing timely interventions and leading to improved outcomes. However, despite providing detailed physiological data, multiple large-scale studies, including randomized controlled trials, have failed to demonstrate clear survival benefits.^(14)^ While PAC provides accurate reflections of hemodynamic derangements, there were no standardized, evidence-based interventions to respond to these findings. Thus, the next step in advancing AI applications is implementing clinical interventions that proactively address predicted risks. Applying robust metrics to evaluate whether these AI-guided interventions effectively mitigate risks, reduce complications, and improve patient outcomes will be equally important.

## CHALLENGES TO WIDESPREAD ADOPTION OF ARTIFICIAL INTELLIGENCE ALGORITHMS

Artificial intelligence adoption faces significant challenges. Standardized and interoperable data formats are essential for implementation across institutions, but data collection and labeling variations hinder model generalizability. To gain trust and facilitate application, AI models must be scalable, reproducible, and explainable, yet many, especially deep learning models, lack transparency. Seamless integration into clinical workflows is critical, as poorly designed interfaces or outputs limit utility even with high accuracy; frameworks like Salient^(13)^ may aid implementation. Bias in training data can exacerbate disparities, raising ethical concerns, particularly when outcomes vary across populations.^(15)^ While promising, AI's performance often only slightly surpasses traditional methods or human clinical judgment. For instance, in predicting postoperative mortality, ML models demonstrated marginal improvements over traditional scoring systems like the American Society of Anesthesiologists (ASA) score, with the Risk Stratification Index still outperforming ML in some cases.^(16)^ Furthermore, studies such as the ORACLE trial have found that human clinicians performed similarly to ML-assisted predictions in some scenarios.^(17)^ With large language models increasingly prevalent in daily workflows, an easy tool and framework for evaluating these needs to be available.^(18)^

## CONCLUSION

**Figure 1:**
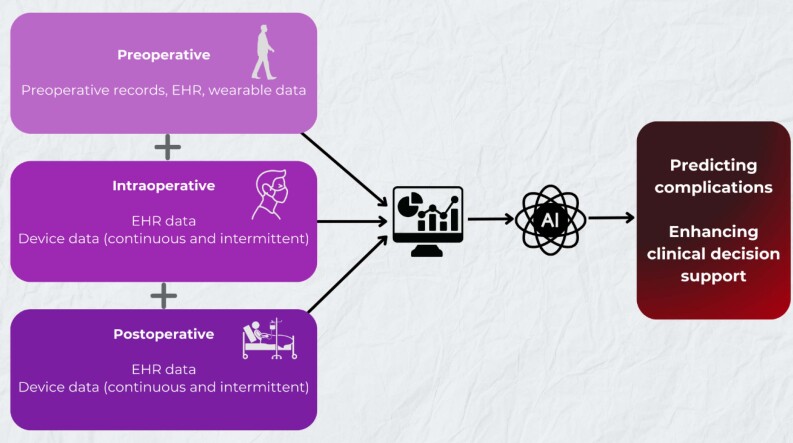
Artificial intelligence models integrate and analyze data from different perioperative phases to predict complications and enhance clinical decision support.

Artificial intelligence and machine learning hold significant potential to transform perioperative care by predicting complications in critically ill patients. These technologies can enhance clinical decision-making by analyzing preoperative, intraoperative, and postoperative data to identify at-risk patients (Figure 1). However, integrating artificial intelligence into clinical practice faces hurdles, including data standardization, model explainability, and ethical concerns. Current models, while promising, have yet to outperform clinical judgment consistently and cannot replace clinical expertise. Instead, they serve as a valuable adjunct, complementing clinicians’ intuition and decision-making. Future efforts should prioritize prospective studies, equitable development, and seamless data integration and transparency to maximize artificial intelligence's benefits. While its promise is clear, a measured and cautious approach is essential to ensure its reliability and accessibility for improving patient outcomes.

EHR - electronic health record; AI - artificial intelligence.
